# Investigation of pathogen resistance in Chinese cancer patients based on bacterial resistance surveillance in 23 provinces, 2016–2023

**DOI:** 10.3389/fmicb.2026.1848531

**Published:** 2026-07-16

**Authors:** Yingnan Ju, Kunbin Liu, Yu Xiao, Gang Ma, Biao Zhu, Hongzhi Wang, Zhenjie Hu, Jianghong Zhao, Li Zhang, Keliang Cui, Xin-rong He, Mingguang Huang, Yang Li, Shanling Xu, Yuetian Yu, Yan Gao, Kaizhong Liu, Guoxing Zhang, Haitao Liu, Yong Ye, Linlin Zhang, Xiangzhe Zhou, Shuliang Ma, Yu’an Qiu, Ming Zhang, Yong Tao, Meiyun Zhang, Lewu Xian, Wei Xie, Guang Wang, Yichun Wang, Changsong Wang, Donghao Wang

**Affiliations:** 1 Department of Critical Care Medicine, Hainan General Hospital (Hainan Affiliated Hospital of Hainan Medical University), Clinical College, Hainan, China; 2 Department of Critical Care Medicine, Tianjin Medical University Cancer Institute and Hospital, Tianjin, China; 3 Department of Critical Care Medicine, The First Affiliated Hospital of Harbin Medical University, Harbin, China; 4 Department of Critical Care Medicine, Sun Yat-sen University Cancer Center, Guangzhou, China; 5 Department of Critical Care Medicine, Fudan University Shanghai Cancer Center, Shanghai, China; 6 Department of Critical Care Medicine, Peking University Cancer Hospital and Institute, Beijing, China; 7 Department of Critical Care Medicine, Hebei Tumor Hospital, Shijiazhuang, China; 8 Department of Critical Care Medicine, Hunan Cancer Hospital, Changsha, China; 9 Department of Critical Care Medicine, Hubei Cancer Hospital, Wuhan, China; 10 Department of Critical Care Medicine, Shanxi Cancer Hospital, Taiyuan, China; 11 Department of Critical Care Medicine, Guangxi Medical University Cancer Hospital, Nanning, China; 12 Department of Critical Care Medicine, Sichuan Cancer Hospital, Chengdu, China; 13 Department of Critical Care Medicine, Renji Hospital, Shanghai Jiao Tong University, School of Medicine, Shanghai, China; 14 Department of Critical Care Medicine, The Fourth Affiliated Hospital of Harbin Medical University, Harbin, China; 15 Department of Critical Care Medicine, Zhejiang Cancer Hospital, Hangzhou, China; 16 Department of Critical Care Medicine, Jilin Provincial Tumor Hospital, Changchun, China; 17 Department of Critical Care Medicine, Harbin Medical University Cancer Hospital, Harbin, China; 18 Department of Critical Care Medicine, Fujian Cancer Hospital, Fuzhou, China; 19 Department of Critical Care Medicine, Anhui Provincial Cancer Hospital, Hefei, China; 20 Department of Critical Care Medicine, Gansu Provincial Cancer Hospital, Lanzhou, China; 21 Department of Critical Care Medicine, Jiangsu Cancer Hospital, Nanjing, China; 22 Department of Critical Care Medicine, Jiangxi Cancer Hospital, Nanchang, China; 23 Department of Critical Care Medicine, Hangzhou Cancer Hospital, Hangzhou, China; 24 Department of Critical Care Medicine, Nantong Tumor Hospital, Nantong, China; 25 Department of Critical Care Medicine, Baotou Cancer Hospital, Baotou, China; 26 Department of Critical Care Medicine, Affiliated Cancer Hospital and Institute of Guangzhou Medical University, Guangzhou, China; 27 Department of Critical Care Medicine, The Affiliated Cancer Hospital of Guizhou Medical University, Guiyang, China; 28 Department of Critical Care Medicine, The First Hospital of Jilin University, Changchun, China; 29 Department of Critical Care Medicine, The Third Affiliated Hospital of Guangzhou Medical University, Guangzhou, China

**Keywords:** antimicrobial stewardship, bacterial resistance, carbapenem resistance, Gram-negative bacteria, Gram-positive bacteria, last-resort antibiotics, multicenter surveillance, tumor

## Abstract

**Background:**

Infection is a common and often fatal complication in cancer patients. Notably, cancer patients account for up to 21% of all sepsis cases. Furthermore, sepsis is present in 30% of all deaths among those hospitalized with cancer. However, comprehensive antimicrobial resistance (AMR) surveillance data specific to this population are lacking. This gap hinders the effectiveness of antimicrobial stewardship programs (ASPs).

**Methods:**

We investigated bacterial distribution and AMR patterns in Chinese cancer patients to inform empirical therapy and ASPs. This multicenter retrospective study collected hospital-wide clinical isolates and drug-susceptibility data from 23 cancer centers during 2016–2023. All procedures followed standardized methods in accordance with national surveillance protocols and Clinical and Laboratory Standards Institute guidelines. Data were subjected to dual validation using WHONET and a domestically developed big-data system.

**Results:**

Gram-negative 75.4% (270,609), Gram-positive 24.6% (88,177) of 358,786 isolates from cancer patients in these oncology centers. Predominant Gram-negative species were *Klebsiella pneumoniae*, *Escherichia coli*, *Pseudomonas aeruginosa*, *Acinetobacter baumannii*, and *Enterobacter cloacae*. *Escherichia coli* exhibited low imipenem resistance (0.9–1.5%) with a recent increase (2021–2023: APC = 16.57%, *p* < 0.001). *Klebsiella pneumoniae* showed stable imipenem resistance (1.6–1.9%) but increasing meropenem resistance (0.4–1.6%). *Pseudomonas aeruginosa* imipenem resistance declined (12.5–7.9%), while meropenem resistance remained stable (7.0–5.2%); while *Acinetobacter baumannii* carbapenem resistance increased substantially (imipenem: 9.6–18.4%; meropenem: 4.7–18.8%). Leading Gram-positive organisms were *Staphylococcus epidermidis*, *Staphylococcus aureus*, *Enterococcus faecium*, *Enterococcus faecalis*, and *Staphylococcus haemolyticus*. Methicillin-resistant *Staphylococcus aureus (MRSA)* resistance declined over five years, with no resistance to linezolid, vancomycin, or teicoplanin. *Enterococcus* spp. gradually developed resistance to last-resort antibiotics.

**Conclusion:**

Carbapenem resistance among common Gram-negatives in cancer patients was below national averages. MRSA and enterococcal resistance were also below national levels. These findings underscore the unique resistance epidemiology in oncology, supporting tailored guidelines and dedicated surveillance.

## 1 Introduction

The global burden of cancer continues to rise and has become a major public health challenge. GLOBOCAN 2022 estimates from the International Agency for Research on Cancer (IARC) reported approximately 20 million new cancer cases and 9.7 million cancer deaths worldwide in 2022, with cancer accounting for nearly one-sixth of all global deaths (Filho et al., 2025). This burden is projected to escalate to 35.3 million new cases and 18.5 million deaths by 2050 (Bizuayehu et al., 2024; Filho et al., 2025). In this global trend, China’s situation is particularly grim. According to the National Cancer Center, there were approximately 4.82 million new cancer cases and 2.57 million cancer deaths in China (Han et al., 2024).

For cancer patients, infections represent a common and often fatal complication. Patients with cancer account for up to 21% of all sepsis cases, and sepsis is present in 30% of all deaths of patients hospitalized with cancer (Nelmes et al., 2023). This vulnerability is compounded by the growing threat of antimicrobial resistance (AMR) in healthcare settings. Immunosuppression, frequent invasive procedures, and prolonged use of broad-spectrum antibiotics make this population particularly susceptible to resistant pathogens. In China, the challenge is especially acute: the detection proportion of multidrug-resistant organisms (MDROs) in cancer centers is substantially higher than in general hospitals (Jiang et al., 2020; Wang et al., 2025), highlighting an urgent need for tailored antimicrobial stewardship programs (ASPs).

Nationwide surveillance systems such as the China Antimicrobial Resistance Surveillance System (CARSS) and the China Antimicrobial Surveillance Network (CHINET) have provided valuable insights into AMR trends in China. However, these systems primarily reflect the epidemiology of general hospital populations and may not accurately represent the unique resistance landscape in oncology settings. Cancer patients differ significantly from the general inpatient population in terms of pathogen distribution, resistance mechanisms, and antibiotic exposure patterns. Furthermore, some literature speculates that the unique tumor microenvironment might have a theoretical association with the horizontal transfer of resistance genes, a hypothesis that could potentially add complexity to treatment (Perry et al., 2022). Despite the recognized severity of AMR in cancer patients, large-scale, long-term systematic surveillance data specifically for the Chinese oncology population remain scarce. Existing studies are often limited by single-center designs, lack of standardized methodologies, or insufficient geographic coverage (Ariza-Heredia and Chemaly, 2018; Boswal et al., 2025). Furthermore, the critical research gap regarding how AMR profiles in cancer patients differ from those in the general hospital population has not been addressed, hindering the development of evidence-based therapy guidelines and effective stewardship strategies tailored to oncology care.

In this study, we conducted the first multicenter AMR surveillance study of cancer patients in China, covering 23 provincial-level regions with an 8-year follow-up period (2016–2023). This retrospective study analyzed microbiological data from 23 top provincial/regional oncology referral centers—all members of CARSS—to systematically delineate the epidemiological characteristics and resistance trends of bacterial pathogens in Chinese cancer patients. Our objectives were to investigate the distribution of clinical specimens, characterize the predominant gram-negative and Gram-positive pathogens, and assess their resistance profiles against commonly used antimicrobial agents. Special emphasis was placed on tracking trends in resistance to key pathogens, including *Escherichia coli (E. coli), Klebsiella pneumoniae (K. pneumoniae), Pseudomonas aeruginosa (P. aeruginosa), Acinetobacter baumannii*, methicillin-resistant *Staphylococcus aureus* (MRSA), and enterococci.

By comparing resistance proportions and microbial compositions over time and across institutions, we aim to provide critical insights for clinicians managing infections in cancer patients, facilitate the rational use of antibiotics, and strengthen infection control strategies. In addition, our study offers a valuable reference for national AMR surveillance efforts, ultimately contributing to the development of more precise antimicrobial stewardship programs tailored to the unique needs of oncology populations.

## 2 Materials and methods

### 2.1 Study design

This retrospective multicenter surveillance study was conducted via a formal data collection notice issued by the Chinese Anti-Cancer Association, collecting clinical bacterial resistance data from 20 provincial cancer diagnosis and treatment centers and 3 specialized cancer hospitals across China during the 8-year period from 2016 to 2023. All participating institutions were the top provincial/regional oncology referral centers in their respective jurisdictions, providing the highest-volume cancer diagnosis and treatment services locally, and all were formal members of CARSS. All study data were derived exclusively from dedicated oncology institutions (cancer centers and specialized cancer hospitals), and the research population consisted of all patients with a pathologically confirmed malignancy admitted to these institutions (across all inpatient wards, not exclusively intensive care units). This cohort represents the broader hospitalized oncology population in China, providing a representative basis for comparison with national surveillance data from general hospitals. Bacterial distribution and AMR data from each participating hospital were systematically collected, collated, standardized, and comparatively analyzed.

### 2.2 Statement of ethical approval

Ethical approval was not required for this study, as it was conducted for public health surveillance purposes using fully anonymized statutory bacterial resistance surveillance data, in strict compliance with the data management guidelines of the National Bacterial Resistance Surveillance Network of China.

### 2.3 Data collection

Data collection was organized and implemented by the Oncology Critical Care Medicine Committee of the Chinese Anti-Cancer Association, via a formal official data collection notice issued to all member units. Prior to data collection, all participating institutions signed a formal written data confidentiality commitment to ensure the security and confidentiality of surveillance data. Bacterial resistance data from 2016 to 2023 were collected from the official CARSS online platform (excluding misspelled CARSS data), including all clinical bacterial isolation results and corresponding antimicrobial susceptibility testing data from each participating institution. Two rounds of standardized data collection were conducted: the first in 2021 for data from 2016 to 2020, and the second in 2024 for data from 2021 to 2023. Only data from institutions that met the full study inclusion criteria were included in the analysis.

### 2.4 Data analysis

Antimicrobial susceptibility testing breakpoint judgments were based on the Clinical and Laboratory Standards Institute (CLSI) M100 Antimicrobial Susceptibility Testing Standards (30th edition) and the latest updated official operational guidelines of CARSS.

Data screening, elimination, and statistical analysis were all conducted in strict accordance with the unified big data analysis standards of CARSS. The core exclusion principles were as follows: Isolates with a drug susceptibility test filling proportion of less than 70% for a single bacterial strain were excluded from statistical analysis; Isolates with a drug susceptibility test breakpoint coverage proportion of less than 90% for a single bacterial strain were excluded from statistical analysis; Duplicate bacterial isolates were rigorously eliminated in accordance with the first-isolate-per-patient principle, defined as retaining only the earliest isolated strain of a specific bacterial species per patient for data analysis. Furthermore, we strictly excluded all active surveillance cultures (such as rectal swabs for carbapenem-resistant enterobacteriaceae (CRE) screening or nasal swabs for MRSA screening). Non-clinical isolates, including laboratory quality control strains, environmental monitoring samples, and research-specific specimens, were also explicitly excluded to ensure that only true clinical diagnostic isolates were analyzed. All data were processed using the Mia Antimicrobial Susceptibility Big-data Operational Analysis System (Version 1.5.88; National Copyright Administration of China, Registration No. 2021SR0329080). To ensure data accuracy and validate this proprietary system, all statistical results—including data screening, isolate counts, and specific resistance proportions—underwent synchronous two-way cross-verification using the internationally recognized WHONET 5.6 software. The parallel processing of the dataset demonstrated complete concordance between the domestically developed system and WHONET, thereby ensuring the reliability of the study data.

Joinpoint regression analysis was performed using the Joinpoint Regression Program (Version 6.0.1; National Cancer Institute, United States) to identify trends in AMR proportions over time. For cohorts with occasional zero values in resistance proportions, a small constant (0.1%) was added to enable log-transformation. The annual percentage change (APC) and corresponding 95% confidence intervals were calculated to assess the significance of trends.

## 3 Results

### 3.1 Overall overview of isolates and main trends

Across 2016–2023, 358,786 isolates were collected from oncology institutions, with Gram-negative bacteria predominating (75.4%) and a modest shift over time toward a higher proportion of Gram-positive bacteria. The most common pathogens were *E. coli* and *K. pneumoniae*, while carbapenem resistance remained low in *E. coli* and *K. pneumoniae* but increased in *Acinetobacter baumannii (A. baumannii)*.

### 3.2 Study sites and data acquisition (two-round collection)

**FIGURE 1:**
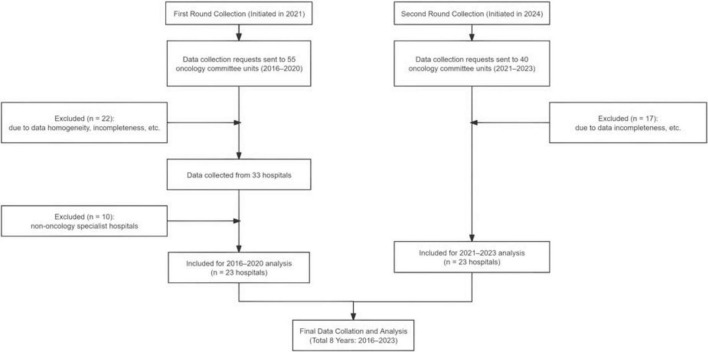
Flow diagram of bacterial resistance data collection in hospitals.

**FIGURE 2:**
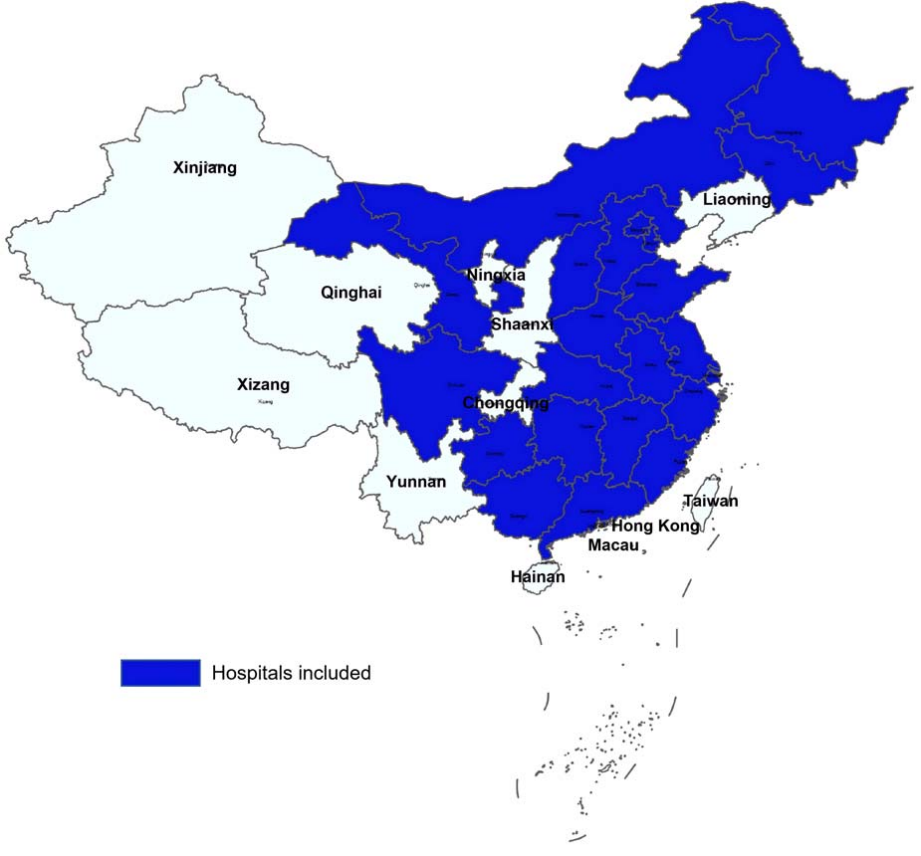
Display the distribution of provinces participating in the research using a map of China, with participating provinces highlighted in blue.

In this study, data were collected from 20 oncology centers and 3 oncology hospitals in 20 provinces. The data for this study were collected twice: the first collection was conducted in 2021, and letters of transfer were sent to 55 hospitals. Data on pathogenic bacteria and their drug resistance across various units from 2016 to 2020 were collected. Information from 41 hospitals in China was collected; 8 were incomplete, and 33 fully met the requirements. Of these, 10 were large general hospitals and non-cancer specialist diagnosis and treatment centers, and were therefore excluded. Finally, information on pathogen drug resistance from 23 hospitals was collected. The second data collection was sent to 40 cancer diagnosis and treatment centers and hospitals in 2024, collecting pathogenic bacteria and drug resistance data from 2021 to 2023, as well as information from 23 hospitals nationwide, all of which fully met the requirements. Then, data on bacterial distribution and drug resistance collected twice over 8 years were collated, compared, and analyzed (Figures 1, 2).

### 3.3 Overall isolate volume and Gram stain distribution (2016–2023)

**FIGURE 3:**
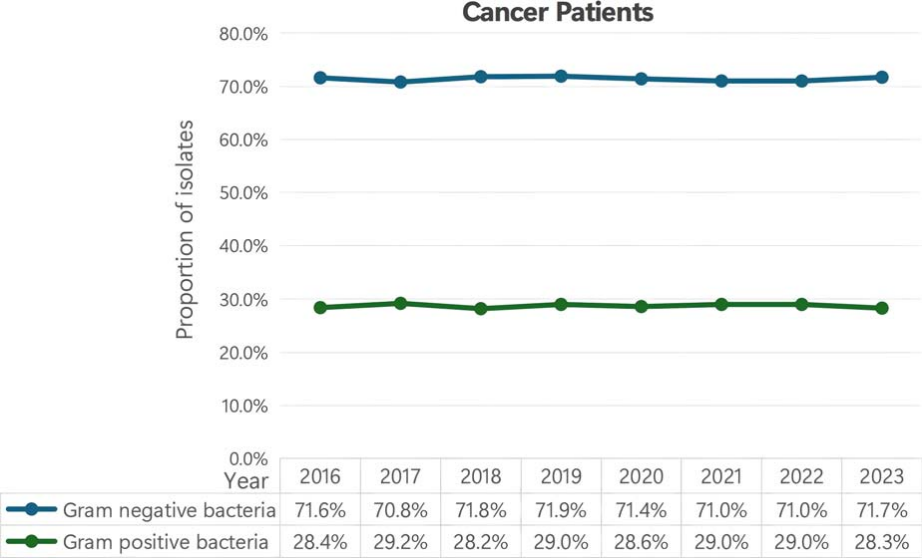
Percentage of Gram-positive bacteria and Gram-negative bacteria in clinical isolates of cancer patients in China.

**FIGURE 4:**
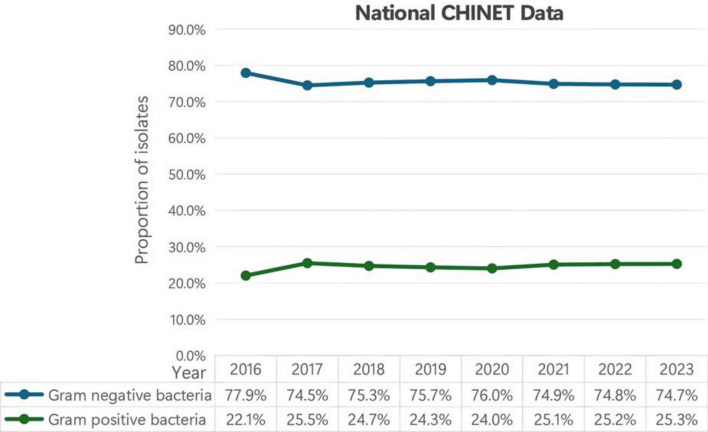
Percentage of Gram-positive cocci and Gram-negative bacilli in clinical isolates in China [data from the China Antibiotic Monitoring Network (CHINET)].

Over the 8-year period from 2016 to 2023, a total of 358,786 bacterial strains were isolated from cancer hospitals. Gram-negative bacteria accounted for 270,609 (75.4%) and Gram-positive bacteria for 88,177 (24.6%), resulting in a Gram-negative to Gram-positive ratio of approximately 7.5:2.5. This ratio is higher than the nationwide hospital average (Figures 3, 4). Throughout this period, the proportion of Gram-negative bacteria declined from 77.9 to 74.7%, while Gram-positive bacteria increased from 22.1 to 25.3%.

### 3.4 Specimen source distribution

**FIGURE 5:**
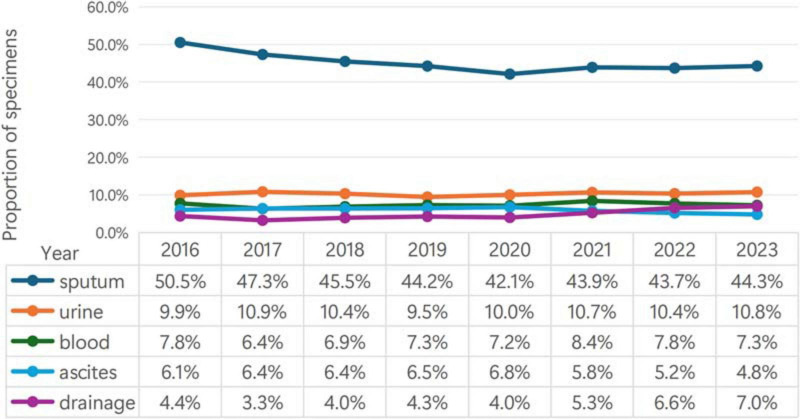
Trends in the proportion of submitted specimens from patients at the cancer hospital (2016–2023).

Across the study period, the proportions of sputum culture sources among clinical isolates in Chinese cancer patients were highest, ranging from 44 to 54%; urine (10–13.0%); blood (7.3–10%); and ascites (7–4.5%). The main specimen sources of patients in China include sputum (36–42%), urine (19–20%), blood (13–15%), and wound pus (7–6.6%). Compared with national data, cancer patients retain more respiratory tract specimens (44–54%) and retain urine and blood specimens in much lower proportions. We can also see that the proportion of respiratory tract specimens retained from cancer patients is decreasing year by year and has been stable over the past 4 years. The proportion of hydrothorax and ascites specimens is higher than national data, which is considered to be related to the disease characteristics of cancer patients (Figure 5).

### 3.5 Top 10 pathogens in cancer patients (2016–2023) and comparison with national surveillance

**FIGURE 6:**
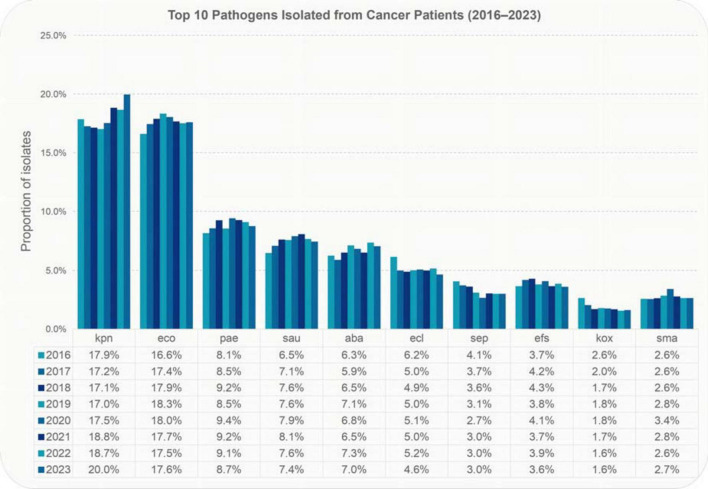
Percentage of the top 10 bacteria isolated from cancer patients. The number represents the percentage of each isolate in all clinical isolates. kpn, *Klebsiella pneumoniae*; eco, *Escherichia coli*; pae, *Pseudomonas aeruginosa*; sau, *Staphylococcus aureus*; aba, *Acinetobacter baumannii*; ecl, *Enterobacter cloacae*; sep, *Staphylococcus epidermidis*; efs, *Enterococcus faecalis*; kox, *Klebsiella oxytoca*; sma, *Stenotrophomonas maltophilia*.

From 2016 to 2023, The 10 most common bacteria in Chinese cancer patients were *E. coli* (16.6–18.3%), *K. pneumoniae* (17–17.9%), *P. aeruginosa* (8.1–9.4%), *Staphylococcus aureus (S. aureus)* (6.5–7.9%), *A. baumannii* (5.9–7.1%), *Enterobacter cloacae (E. cloacae)* (4.9–6.2%), *Staphylococcus epidermidis (S. epidermidis)* (2.7–4.1%), *Enterococcus faecalis (E. faecalis)* (3.7–4.3%), *Klebsiella oxytoca* (1.6–2.6%) and *Stenotrophomonas maltophilia* (2.6–3.4%) (Figure 6). According to the Joinpoint regression analysis (Table 1), the isolation proportion of *E. coli* showed a significant upward trend from 2016 to 2019 (APC = 3.01%, 95% CI: 1.47–6.16, *P* < 0.001), followed by a significant decline during 2019–2023 (APC = −1.27%, 95% CI: −3.29 to −0.31, *P* = 0.007). Conversely, *K. pneumoniae* remained stable between 2016 and 2019 (APC = −1.43%, 95% CI: −4.21 to 0.52, *P* = 0.156), but experienced a significant increase over the subsequent 4 years (APC = 4.03%, 95% CI: 2.77–6.57, *P* < 0.001). We also examined changes in the 10 most common strains in CARSS and CHINET during 2016–2020. *Streptococcus pneumoniae* and *Streptococcus hemolyticus B* ranked among the top 10 strains in CARSS, but ranked 23rd and 40th in cancer patients, respectively.

**TABLE 1:** Joinpoint regression analysis of temporal trends for the major isolated pathogens.

Pathogen	Initial year (%)	Final year (%)	Trend Period	APC (%) (95% CI)	*P*-value	Trend
*Escherichia coli*	16.61	17.59	2016–2019	3.01* (1.47, 6.16)	<0.001	Increasing
			2019–2023	−1.27* (−3.29, −0.31)	0.007	Decreasing
*Klebsiella pneumoniae*	17.85	19.96	2016–2019	−1.43 (−4.21, 0.53)	0.156	Stable
			2019–2023	4.03* (2.77, 6.57)	<0.001	Increasing
Pseudomonas aeruginosa	8.14	8.74	2016–2020	3.13* (1.05, 8.16)	0.019	Increasing
			2020–2023	−1.87 (−6.73, 0.94)	0.201	Stable
*Staphylococcus aureus*	6.48	7.42	2016–2020	5.03* (2.83, 12.70)	0.005	Increasing
			2020–2023	−2.61 (−10.26, 0.61)	0.122	Stable
*Acinetobacter baumannii* *Enterobacter cloacae*	6.25 6.16	7.04 4.65	2016–2023	2.26* (0.10, 4.51)	0.038	Increasing
			2016–2018	−8.84* (−14.18, −1.93)	0.002	Decreasing
			2018–2023	0.06 (−2.79, 6.10)	0.614	Stable
*Staphylococcus epidermidis*	4.07	3.00	2016–2020	−9.40* (−14.56, −6.81)	<0.001	Decreasing
			2020–2022	4.42 (−3.11, 10.58)	0.324	Stable
*Enterococcus faecalis*	3.65	3.61	2016–2018	5.94 (−3.21, 16.76)	0.199	Stable
			2018–2023	−3.02 (−12.30, 2.09)	0.129	Stable
*Klebsiella oxytoca*	2.64	1.62	2016–2018	−17.74* (−23.17, −8.12)	<0.001	Decreasing
			2018–2023	−1.64 (−4.68, 6.89)	0.569	Stable
*Stenotrophomonas maltophilia*	2.57	2.65	2016–2020	6.11* (1.99, 16.94)	0.010	Increasing
			2020–2023	−6.36* (−16.30, −0.81)	0.023	Decreasing

The final year refers to 2023 for most pathogens, except for *S. epidermidis*, for which it refers to 2022, consistent with their respective trend periods. APC, annual percent change; CI, confidence interval.

* Indicates that the APC is significantly different from zero at the alpha = 0.05 level.

### 3.6 Distribution trends of major Gram-negative species

**FIGURE 7:**
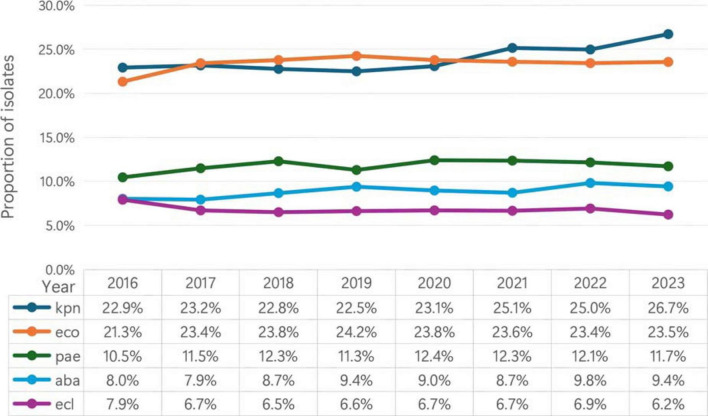
Percentage of the top five Gram-negative bacteria. The number represents the percentage of each isolate in all Gram-negative clinical isolates. kpn, *Klebsiella pneumoniae*; eco, *Escherichia coli*; pae, *Pseudomonas aeruginosa*; aba, *Acinetobacter baumannii*; ecl, *Enterobacter cloacae*.

Throughout the surveillance period, *K. pneumoniae* and *E. coli* were the most prevalent Gram-negative bacteria, accounting for 22.91–26.71% and 21.31–23.55% of Gram-negative isolates, respectively (Figure 7). According to the Joinpoint regression analysis (Table 2), the isolation proportion of *K. pneumoniae* remained stable from 2016 to 2019 (APC = −0.84%, 95% CI: −4.09 to 1.35, *P* = 0.415), followed by a significant increase during 2019–2023 (APC = 4.21%, 95% CI: 2.71 to 7.65, *P* < 0.001). For *E. coli*, a significant upward trend was observed between 2016 and 2018 (APC = 5.77%, 95% CI: 2.20–9.79, *P* < 0.001), after which the proportion remained stable through 2023 (APC = −0.62%, 95% CI: −2.77 to 0.17, *P* = 0.112). *P. aeruginosa* comprised 10.45%–11.70% of the isolates, exhibiting a significant increase from 2016 to 2018 (APC = 7.09%, 95% CI: 2.01–12.63, *P* = 0.002), followed by a stable trend over the subsequent 5 years (APC = −0.18%, 95% CI: −4.66 to 1.39, *P* = 0.605). *A. baumannii* accounted for 8.02%–9.42% of Gram-negative bacteria and showed a significant steady increase throughout the entire study period (2016–2023: APC = 2.61%, 95% CI: 0.14–5.18, *P* = 0.039). In contrast, *E. cloacae*, which accounted for 7.91% in 2016 and 6.22% in 2023, showed a significant decreasing trend during 2016–2018 (APC = −7.79%, 95% CI: −12.14 to −2.06, *P* < 0.001), followed by a stable phase through 2023 (APC = 0.15%, 95% CI: −1.91 to 5.02, *P* = 0.569).

**TABLE 2:** Joinpoint regression analysis of temporal trends for the major isolated Gram-negative pathogens.

Pathogen	Initial year (%)	Final year (%)	Trend Period	APC (%) (95% CI)	*P*-value	Trend
*Klebsiella pneumoniae*	22.91	26.71	2016–2019	−0.84 (−**4**.09, 1.35)	0.415	Stable
			2019–2023	4.21* (2.71, 7.65)	<0.001	Increasing
*Escherichia coli*	21.31	23.55	2016–2018	5.77* (2.20, 9.79)	<0.001	Increasing
			2018–2023	−0.62 (−2.77, 0.17)	0.112	Stable
*Pseudomonas aeruginosa*	10.45	11.70	2016–2018	7.09* (2.01, 12.63)	0.002	Increasing
			2018–2023	−0.18 (−4.66, 1.39)	0.605	Stable
*Acinetobacter baumannii*	8.02	9.42	2016–2023	2.61* (0.14, 5.18)	0.039	Increasing
*Enterobacter cloacae*	7.91	6.22	2016–2018	−7.79* (−12.14, −2.06)	<0.001	Decreasing
			2018–2023	0.15 (−1.91, 5.02)	0.569	Stable

APC, annual percent change; CI, confidence interval.

* Indicates that the APC is significantly different from zero at the alpha = 0.05 level.

### 3.7 Distribution trends of major Gram-positive species

**FIGURE 8:**
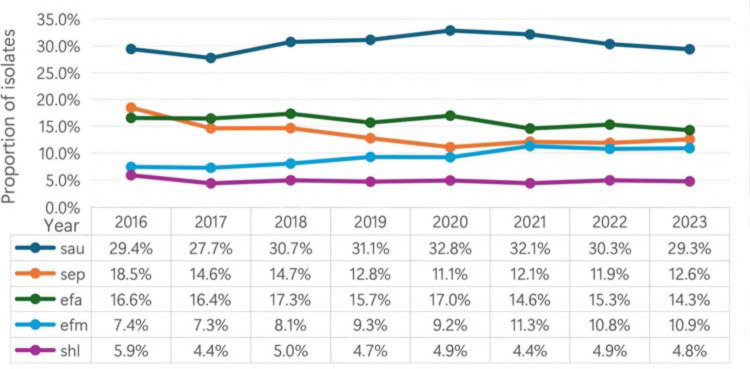
Percentage of the top five Gram-positive bacteria. The number represents the percentage of each isolate in all Gram-positive clinical isolates. sau, *Staphylococcus aureus*; sep, *Staphylococcus epidermidis*; efa, *Enterococcus faecalis*; efm, *Enterococcus faecium*; shl, *Staphylococcus haemolyticus*.

During 2016–2023, the top five Gram-positive bacteria isolated from cancer patients in 2016–2023 were *S. aureus* (27.1–32.8%), *E. faecalis* (14.3–17.3%), *S. epidermidis* (11.1–18.5%), *Enterococcus faecium (E. faecium)* (7.3–11.3%), and *Staphylococcus haemolyticus (S. haemolyticus)* (4.4–5.9%) (Figure 8). According to the Joinpoint regression analysis (Table 3), *S. aureus* showed a significant upward trend from 2016 to 2020 (APC = 5.03%, 95% CI: 2.83–12.70, *P* = 0.005), followed by a stable trend over the last 3 years (2020–2023: APC = −2.61%, 95% CI: −10.26 to 0.61, *P* = 0.122). Detection of *S. epidermidis* decreased significantly between 2016 and 2020 (APC = −9.40%, 95% CI: −14.56 to −6.81, *P* < 0.001), then remained stable during 2020–2022 (APC = 4.42%, 95% CI: −3.11 to 10.58, *P* = 0.324). The isolation proportion of *E. faecalis* remained statistically stable throughout the period, both during 2016–2018 (APC = 5.94%, 95% CI: −3.21 to 16.76, *P* = 0.199) and 2018–2023 (APC = −3.02%, 95% CI: −12.30 to 2.09, *P* = 0.129). On the contrary, *E. faecium* experienced a significant increase from 2016 to 2021 (APC = 10.26%, 95% CI: 6.40–18.59, *P* < 0.001), before stabilizing in the subsequent years (2021–2023: APC = 1.43%, 95% CI: −5.72 to 9.12, *P* = 0.440). The trends for *S. haemolyticus* (2016–2022: APC = −0.67%, 95% CI: −6.34 to 5.58, *P* = 0.754) and *Staphylococcus hominis* (2016–2022: APC = 3.15%, 95% CI: −2.84 to 9.66, *P* = 0.288) were stable over the study period.

**TABLE 3:** Joinpoint regression analysis of temporal trends for the major isolated Gram-positive pathogens.

Pathogen	Initial year (%)	Final year (%)	Trend period	APC (%) (95% CI)	*P*—value	Trend
*Staphylococcus aureus*	29.38	29.33	2016–2020	3.64* (1.85, 7.52)	<0.001	Increasing
			2020–2023	−3.37* (−7.91, −0.72)	0.004	Decreasing
*Staphylococcus epidermidis*	18.46	12.58	2016–2020	−10.58* (−17.77, −7.00)	<0.001	Decreasing
			2020–2023	3.50 (−2.58, 13.41)	0.287	Stable
*Enterococcus faecalis* *Enterococcus faecium*	16.55 7.44	14.28 10.92	2016–2023	−2.15* (−4.14, −0.07)	0.044	Decreasing
			2016–2023	6.98* (3.24, 10.90)	<0.001	Increasing
*Staphylococcus haemolyticus*	5.89	4.75	2016–2023	−1.42 (−4.72, 2.04)	0.416	Stable

APC, annual percent change; CI, confidence interval.

*Indicates that the APC is significantly different from zero at the alpha = 0.05 level.

### 3.8 Antimicrobial resistance patterns in key Gram-negative pathogens

#### 3.8.1 Escherichia coli

As detailed in Table 4, over the 8-year period, *E. coli* exhibited relatively high resistance proportions to most cephalosporins and quinolones, typically ranging from 50 to 60%. Based on the Joinpoint regression analysis (Table 5), resistance to ceftazidime ranged from 22.6 to 27.6%, showing a significant downward trend (APC = −2.31%, 95% CI: −3.95 to −0.60, *P* = 0.009) and remaining considerably lower than that observed for cefotaxime. In contrast, resistance to ciprofloxacin remained statistically stable over the study period, fluctuating between 54.3 and 58.4% (2016–2018: APC = −1.57%, 95% CI: −3.55 to 1.26, *P* = 0.250; 2018–2023: APC = 1.38%, 95% CI: −0.40 to 3.86, *P* = 0.057). Resistance to piperacillin-tazobactam showed a significant increase, rising from 3.2% in 2016 to 6.5% in 2023, with a peak of 7.2% in 2021 (APC = 14.64%, 95% CI: 1.86–29.17, *P* = 0.026). Imipenem resistance in *E. coli* was the lowest among the tested antibiotics, ranging from 0.9 to 1.5%. While it remained stable from 2016 to 2021 (APC = 4.09%, 95% CI: −4.60 to 9.83, *P* = 0.328), it exhibited a significant upward trend between 2021 and 2023 (APC = 16.57%, 95% CI: 5.24–26.23, *P* < 0.001), though it stayed far below the national average.

**TABLE 4:** Resistance proportions (%) of *Escherichia coli* to antimicrobial agents.

Antimicrobial agent	2016	2017	2018	2019	2020	2021	2022	2023
AMK	3.3	2.9	2.9	2.7	3.2	2.6	2.4	2.4
GEN	43.7	43.2	41.6	40.6	39.1	38.3	37.3	38.3
TOB	19.9	18.3	14.9	14.2	13.3	12.1	11.7	13.6
AMP	87.2	86.0	85.8	85.7	87.0	85.3	84.0	85.4
AMC	9.9	9.1	9.0	10.7	10.7	11.4	11.8	11.3
TCC	8.5	9.8	10.2	11.4	8.3	12.4	12.7	8.8
SAM	47.4	45.8	41.4	39.0	35.6	36.1	36.5	35.3
TZP	3.2	3.2	2.9	3.7	3.5	7.2	6.9	6.5
CSL	2.9	5.0	2.6	5.3	6.4	3.6	5.0	6.4
CZO	82.8	81.5	78.5	74.4	76.2	72.5	74.0	76.4
CXM	60.3	59.9	55.3	58.1	61.0	57.9	55.8	60.3
CRO	62.1	60.0	57.6	58.6	59.3	56.1	55.6	59.0
CTX	53.3	55.4	54.6	54.0	53.1	56.1	52.6	55.1
CAZ	27.6	27.1	23.7	25.3	24.3	23.9	22.6	23.8
FEP	23.0	25.9	25.3	27.7	28.5	29.1	27.9	30.2
CTT	2.1	1.7	1.9	1.4	1.4	2.2	1.7	1.8
FOX	11.9	12.4	12.0	13.8	13.3	11.4	13.2	13.8
ATM	37.3	39.2	36.2	36.9	38.0	34.5	34.6	36.3
IPM	0.9	0.9	0.9	1.1	1.0	1.1	1.2	1.5
MEM	0.9	0.8	0.7	1.0	0.9	1.4	1.5	1.7
ETP	0.5	0.4	0.5	0.8	0.9	0.7	1.1	1.7
CIP	56.6	56.3	54.3	55.5	57.6	57.9	57.9	58.4
LVX	53.0	52.5	51.4	52.6	54.0	53.6	53.8	54.3
SXT	60.2	57.8	57.8	58.9	59.3	58.1	55.3	55.9
CHL	37.7	41.1	39.2	37.9	40.9	37.7	35.4	35.5
TGC	0.0	0.0	0.1	0.1	0.1	0.0	0.1	0.2
POL						1.1	1.2	0.4

AMK, amikacin; GEN, gentamicin; TOB, tobramycin; AMC, amoxicillin-clavulanic acid; TCC, ticarcillin-clavulanic acid; SAM, ampicillin-sulbactam; TZP, piperacillin-tazobactam; CSL, cefoperazone-sulbactam; CZO, cefazolin; CXM, cefuroxime; CRO, ceftriaxone; CTX, cefotaxime; CAZ, ceftazidime; FEP, cefepime; CTT, cefotetan; FOX, cefoxitin; ATM, aztreonam; IPM, imipenem; MEM, meropenem; ETP, ertapenem; CIP, ciprofloxacin; LVX, levofloxacin; SXT, trimethoprim-sulfamethoxazole; CHL, chloramphenicol; TGC, tigecycline; POL, polymyxin B.

**TABLE 5:** Joinpoint regression analysis of temporal trends for key antimicrobial resistance indicators in major pathogens.

Pathogen and antimicrobial agent	Trend description (2016–2023)
1. Escherichia coli	
Piperacillin-tazobactam	Increasing: 3.20–6.50% (APC = 14.64%*, 95% CI: 1.86–29.17, *P* = 0.026)
Cefotaxime	Stable: 53.30–55.10% (APC = 0.04%, 95% CI: −1.30 to 1.44, *P* = 0.928)
Ceftazidime	Decreasing: 27.60–23.80% (APC = −2.31%*, 95% CI: −3.95 to −0.60, *P* = 0.009)
Imipenem	Stable then increasing: Stable from 2016 to 2021 (APC = 4.09%, 95% CI: −4.60 to 9.83, *P* = 0.328), followed by a significant increasing trend from 2021 to 2023 (APC = 16.57%*, 95% CI: 5.24–26.23, *P* < 0.001). Overall: 0.90–1.50%.
Ciprofloxacin	Stable Stable from 2016 to 2018 (APC = −1.57%, 95% CI: −3.55 to 1.26, *P* = 0.250), and stable from 2018 to 2023 (APC = 1.38%, 95% CI: −0.40 to 3.86, *P* = 0.057). Overall: 56.60–58.40%.
2. Klebsiella pneumoniae	
Piperacillin-tazobactam	Increasing: 3.00–7.10% (APC = 15.49%*, 95% CI: 7.71–23.85, *P* < 0.001)
Cefoperazone-sulbactam	Stable: 3.60–5.80% (APC = 5.02%, 95% CI: −8.06 to 20.50, *P* = 0.450)
Cefotaxime	Stable: 7.90–12.60% (APC = 1.61%, 95% CI: −3.99 to 7.49, *P* = 0.581)
Imipenem	Stable: 1.60–1.90% (APC = 1.64%, 95% CI: −1.67 to 5.08, *P* = 0.352)
Meropenem	Increasing: 0.40–1.60% (APC = 19.09%*, 95% CI: 0.80–41.37, *P* = 0.038)
3. Pseudomonas aeruginosa	
Piperacillin-tazobactam	Stable: 5.30–4.40% (APC = −3.04%, 95% CI: −9.37 to 3.74, *P* = 0.331)
Cefoperazone-sulbactam	Stable: 1.90–6.60% (APC = 9.81%, 95% CI: −1.06 to 21.73, *P* = 0.074)
Ceftazidime	Stable: 5.90–8.00% (APC = 2.74%, 95% CI: −3.14 to 9.02, *P* = 0.313)
Imipenem	Decreasing then Stable Decreasing from 2016 to 2020 (APC = −10.96%*, 95% CI: −15.91 to −8.47, *P* < 0.001), followed by a Stable trend from 2020 to 2023 (APC = −0.47%, 95% CI: −4.81 to 6.33, *P* = 0.878). Overall: 12.50–7.90%.
Meropenem	Stable: 7.00–5.20% (APC = −4.09%, 95% CI: −8.06 to 0.12, *P* = 0.060)
Levofloxacin	Stable: 5.80–6.00% (APC = 2.66%, 95% CI: −0.25 to 5.77, *P* = 0.080)
4. Acinetobacter baumannii	
Cefoperazone-sulbactam	Stable: 22.80–20.40% (APC = 6.71%, 95% CI: −10.13 to 27.05, *P* = 0.444)
Imipenem	Increasing: 9.60–18.40% (APC = 7.75%*, 95% CI: 2.50–13.33, *P* = 0.006)
Meropenem	Increasing then Stable Increasing from 2016 to 2019 (APC = 37.85%*, 95% CI: 21.59–86.36, *P* < 0.001), followed by a Stable trend from 2019 to 2023 (APC = 7.59%, 95% CI: −18.52 to 17.45, *P* = 0.309). Overall: 4.70–18.80%.
5. MRSA	
Penicillin	Stable: 99.50–100.00% (APC = 0.14%, 95% CI: −0.03 to 0.31, *P* = 0.102)
Levofloxacin	Decreasing: 30.20–19.30% (APC = −7.40%*, 95% CI: −10.48 to −4.15, *P* < 0.001)
Trimethoprim-sulfamethoxazole	Decreasing then Stable Decreasing from 2016 to 2019 (APC = −20.27%*, 95% CI: −35.90 to −8.50, *P* = 0.004), followed by a Stable trend from 2019 to 2023 (APC = 5.11%, 95% CI: −4.42 to 29.61, *P* = 0.290). Overall: 16.60–9.50%.
Clindamycin	Stable Stable from 2016 to 2019 (APC = −7.25%, 95% CI: −17.04 to 3.29, *P* = 0.099), and stable from 2019 to 2023 (APC = 0.77%, 95% CI: −9.01 to 12.71, *P* = 0.556). Overall: 65.20–51.20%.
Erythromycin	Decreasing then Stable Decreasing from 2016 to 2019 (APC = −5.41%*, 95% CI: −9.71 to −2.14, *P* = 0.003), followed by a Stable trend from 2019 to 2023 (APC = −1.11%, 95% CI: −3.71 to 3.67, *P* = 0.653). Overall: 83.40–66.50%.
6. Enterococcus faecium	
Ciprofloxacin	Decreasing then Stable Decreasing from 2016 to 2019 (APC = −2.57%*, 95% CI: −5.93 to −0.08, *P* = 0.040), followed by a Stable trend from 2019 to 2023 (APC = 1.55%, 95% CI: −0.15 to 5.21, *P* = 0.076). Overall: 82.40–82.10%.
Ampicillin	Stable Stable from 2016 to 2020 (APC = −0.91%, 95% CI: −3.54 to 0.46, *P* = 0.139), and stable from 2020 to 2023 (APC = 1.83%, 95% CI: −0.05 to 4.64, *P* = 0.057). Overall: 81.80–81.70%.
Levofloxacin	Decreasing then Increasing Decreasing from 2016 to 2019 (APC = −2.07%*, 95% CI: −3.90 to −0.91, *P* < 0.001), followed by a significant increasing trend from 2019 to 2023 (APC = 2.04%*, 95% CI: 1.29–3.39, *P* < 0.001). Overall: 79.50–81.30%.
Gentamicin high level	Stable: 21.20–33.70% (APC = 7.08%, 95% CI: −2.19 to 17.37, *P* = 0.146)
Streptomycin high level	Increasing then stable Increasing from 2016 to 2018 (APC = 41.45%*, 95% CI: 12.84–74.02, *P* < 0.001), followed by a Stable trend from 2018 to 2023 (APC = −2.80%, 95% CI: −16.42 to 2.51, *P* = 0.236). Overall: 13.20–22.90%.
Linezolid	Stable Stable from 2016 to 2018 (APC = −69.78%, 95% CI: −89.14 to 25.60, *P* = 0.128), and stable from 2018 to 2023 (APC = 51.57%, 95% CI: −15.19 to 416.19, *P* = 0.092). Overall: 1.90–0.70%.
Vancomycin	Stable Stable from 2016 to 2020 (APC = −18.11%, 95% CI: −70.93 to 98.89, *P* = 0.278), and stable from 2020 to 2023 (APC = 93.07%, 95% CI: −19.17 to 438.32, *P* = 0.084). Overall: 0.50–2.20%.
Teicoplanin	Stable: 0.00–1.40% (APC = 29.67%, 95% CI: −18.37 to 108.64, *P* = 0.253)
7. Enterococcus faecalis	
Ciprofloxacin	Stable Stable from 2016 to 2021 (APC = 8.23%, 95% CI: −0.43 to 26.30, *P* = 0.060), and stable from 2021 to 2023 (APC = −3.41%, 95% CI: −16.96 to 10.11, *P* = 0.755). Overall: 27.40–36.80%.
Levofloxacin	Stable: 24.10% to 28.40% (APC = 2.46%, 95% CI: −1.41 to 6.50, *P* = 0.191)
Gentamicin high level	Increasing: 16.20% to 28.70% (APC = 8.78%*, 95% CI: 6.23 to 11.43, *P* < 0.001)
Ampicillin	Stable: 3.80% to 2.80% (APC = 0.16%, 95% CI: −10.60 to 12.57, *P* = 0.969)
Linezolid	Stable: 3.80–3.40% (APC = 1.67%, 95% CI: −9.23 to 14.14, *P* = 0.721)
Teicoplanin	Stable: 2.90–0.40% (APC = −17.30%, 95% CI: −44.31 to 24.28, *P* = 0.370)
Vancomycin	Stable Stable from 2016 to 2018 (APC = −21.96%, 95% CI: −45.08 to 20.29, *P* = 0.348), and stable from 2018 to 2023 (APC = 21.87%, 95% CI: −8.50 to 77.90, *P* = 0.081). Overall: 0.20–0.40%.

APC, annual percent change; CI, confidence interval. *Indicates that the APC is significantly different from zero at the alpha = 0.05 level.

#### 3.8.2 Klebsiella pneumoniae

Throughout the study period, as detailed in Table 6, *K. pneumoniae* showed relatively low resistance to most antibiotics tested, though increasing trends were observed for several agents. Based on the Joinpoint regression analysis (Table 5), resistance to cefotaxime fluctuated between 7.9 and 12.6% but remained statistically stable (APC = 1.61%, 95% CI: −3.99 to 7.49, *P* = 0.581), while quinolone resistance remained stable. Piperacillin-tazobactam resistance significantly increased from 3.0 to 7.1% (APC = 15.49%, 95% CI: 7.71–23.85, *P* < 0.001), whereas cefoperazone-sulbactam resistance remained statistically stable despite a numerical rise from 3.6 to 5.8% (APC = 5.02%, 95% CI: −8.06 to 20.50, *P* = 0.450). Among carbapenems, meropenem resistance rose markedly significantly from 0.4 to 1.6% (APC = 19.09%, 95% CI: 0.80–41.37, *P* = 0.038), whereas imipenem resistance remained stable (1.6–1.9%) (APC = 1.64%, 95% CI: −1.67 to 5.08, *P* = 0.352). Despite these increases, carbapenem resistance in *K. pneumoniae* remained well below the national average of 16–27%.

**TABLE 6:** Resistance proportions (%) of *Klebsiella pneumoniae* to antimicrobial agents.

Antimicrobial agent	2016	2017	2018	2019	2020	2021	2022	2023
AMK	1.9	1.8	1.9	1.6	2.0	2.0	1.7	1.8
GEN	10.4	11.8	11.3	9.9	10.3	9.8	8.1	9.1
TOB	6.8	6.8	5.5	4.5	4.2	4.3	3.2	5.2
AMC	5.3	5.2	5.8	6.2	8.9	10.6	6.4	6.6
TCC	3.2	3.8	7.4	5.8	7.4	6.1	5.3	4.9
SAM	13.5	17.0	17.1	15.9	18.4	18.1	16.2	16.2
TZP	3.0	3.0	3.3	3.2	3.9	6.6	6.4	7.1
CSL	3.6	3.1	2.0	3.9	4.3	2.8	2.9	5.8
CZO	26.9	28.3	24.3	23.8	29.4	24.4	23.5	26.9
CXM	14.8	17.2	15.1	16.1	18.5	17.1	13.8	18.3
CRO	14.3	18.0	17.2	17.4	19.8	15.7	13.4	16.7
CTX	7.9	13.0	12.2	11.1	12.4	11.0	9.2	12.6
CAZ	7.3	7.8	7.5	8.5	9.4	8.7	8.6	9.7
FEP	5.4	8.9	8.6	9.2	9.7	10.0	8.9	10.2
CTT	1.2	1.5	1.7	0.8	0.6	2.5	1.7	1.0
FOX	7.9	7.5	8.4	9.2	8.9	7.1	7.7	9.7
ATM	9.8	12.9	12.1	11.8	12.4	11.7	8.8	12.9
IPM	1.6	1.9	1.8	1.5	1.5	1.9	1.9	1.9
MEM	0.4	1.2	0.9	0.6	1.0	1.7	2.0	1.6
ETP	0.6	0.5	0.5	0.8	1.0	1.0	0.8	1.6
CIP	9.9	11.1	10.9	10.5	11.0	9.7	8.4	10.1
LVX	7.4	8.6	7.8	8.4	8.5	7.8	7.2	8.7
SXT	17.4	18.3	19.5	18.6	20.1	17.2	15.7	17.1
CHL	23.1	24.0	23.9	19.7	22.7	21.0	19.9	19.3
TGC	0.3	1.1	0.6	1.2	1.8	1.6	1.3	1.7
POL						2.2	2.7	1.2

AMK, amikacin; GEN, gentamicin; TOB, tobramycin; AMP, ampicillin; AMC, amoxicillin-clavulanic acid; TCC, ticarcillin-clavulanic acid; SAM, ampicillin-sulbactam; TZP, piperacillin-tazobactam; CSL, cefoperazone-sulbactam; CZO, cefazolin; CXM, cefuroxime; CRO, ceftriaxone; CTX, cefotaxime; CAZ, ceftazidime; FEP, cefepime; CTT, cefotetan; FOX, cefoxitin; ATM, aztreonam; IPM, imipenem; MEM, meropenem; ETP, ertapenem; CIP, ciprofloxacin; LVX, levofloxacin; SXT, trimethoprim-sulfamethoxazole; CHL, chloramphenicol; TGC, tigecycline; POL, polymyxin B.

#### 3.8.3 Pseudomonas aeruginosa

From 2016 to 2023, as shown in Table 7, *P. aeruginosa* exhibited statistically stable resistance proportions to nearly all commonly used antibiotics, except for a significant decline in imipenem resistance during the 2016–2020 period. According to the Joinpoint regression analysis (Table 5), susceptibility to fluoroquinolones [e.g., ciprofloxacin and levofloxacin (APC = 2.66%, 95% CI: −0.25 to 5.77, *P* = 0.080) and piperacillin-tazobactam (APC = −3.04%, 95% CI: −9.37 to 3.74, *P* = 0.331)] remained relatively high, and resistance levels remained statistically stable throughout the study period. Regarding cefoperazone-sulbactam (APC = 9.81%, 95% CI: −1.06 to 21.73, *P* = 0.074) and ceftazidime (APC = 2.74%, 95% CI: −3.14 to 9.02, *P* = 0.313), resistance also showed no significant change. Regarding carbapenems, resistance to imipenem significantly declined from 12.5% in 2016 to 7.9% by 2020 (2016–2020: APC = −10.96%, 95% CI: −15.91 to −8.47, *P* < 0.001) and then remained stable through 2023 (2020–2023: APC = −0.47%, 95% CI: −4.81 to 6.33, *P* = 0.878), while resistance to meropenem remained statistically stable, despite a numerical decrease from 7.0 to 5.2% (APC = −4.09%, 95% CI: −8.06 to 0.12, *P* = 0.060) over the same period. Nationally, carbapenem resistance in *P. aeruginosa* also showed a downward trend, ranging from 17 to 31%, yet remained substantially higher than the levels observed in cancer hospitals.

**TABLE 7:** Resistance proportions (%) of *Pseudomonas aeruginosa* to antimicrobial agents.

Antimicrobial agent	2016	2017	2018	2019	2020	2021	2022	2023
AMK	2.0	3.2	1.9	1.2	1.6	1.3	0.7	1.1
GEN	6.2	5.3	4.5	3.5	3.8	3.5	3.2	3.1
TOB	4.7	3.0	2.7	2.2	1.3	1.9	1.5	1.7
PIP	7.8	6.8	5.4	6.4	6.7	9.5	7.7	7.1
TZP	5.3	5.6	6.2	6.2	4.3	5.0	5.3	4.4
CSL	1.9	5.9	3.7	4.2	5.0	3.7	4.8	6.6
CAZ	5.9	7.9	6.6	8.1	6.6	7.9	7.6	8.0
FEP	5.9	6.7	5.8	4.9	3.7	5.4	5.0	4.2
ATM	15.0	14.4	17.4	14.5	13.5	14.6	14.4	13.1
IPM	12.5	10.7	9.9	8.8	7.7	8.0	7.2	7.9
MEM	7.0	6.6	6.6	4.6	5.1	5.9	5.2	5.2
CIP	6.7	4.7	5.3	5.4	4.6	5.4	5.6	4.8
LVX	5.8	4.6	5.6	6.0	5.8	6.3	6.4	6.0
POL	4.4	0.2	1.0	0.4	0.3	4.3	2.5	4.2

AMK, amikacin; GEN, gentamicin; TOB, tobramycin; PIP, piperacillin; TZP, piperacillin-tazobactam; CSL, cefoperazone-sulbactam; CAZ, ceftazidime; FEP, cefepime; ATM, aztreonam; IPM, imipenem; MEM, meropenem; CIP, ciprofloxacin; LVX, levofloxacin; POL, polymyxin B.

#### 3.8.4 Acinetobacter baumannii

Over the 2016–2023 period, as detailed in Table 8, *A. baumannii* exhibited increasing resistance to nearly all commonly used antibiotics, except polymyxin, minocycline, and tigecycline. Based on the Joinpoint regression analysis (Table 5), this upward trend was particularly pronounced for carbapenems: imipenem resistance rose from 9.6% in 2016 to 18.4% in 2023 (APC = 7.75%, 95% CI: 2.50–13.33, *P* = 0.006), while meropenem resistance experienced a significant surge between 2016 and 2019 (2016–2019: APC = 37.85%, 95% CI: 21.59–86.36, *P* < 0.001), after which it remained statistically stable, reaching 18.8% in 2023 (2019–2023: APC = 7.59%, 95% CI: −18.52 to 17.45, *P* = 0.309). Resistance to cefoperazone-sulbactam showed a fluctuating pattern—starting at 22.8% in 2016, dropping to 4.2% in 2017, then rising to 12.9% in 2022—but remained statistically stable overall across the study period (APC = 6.71%, 95% CI: −10.13 to 27.05, *P* = 0.444). Despite these increases, carbapenem resistance in *A. baumannii* from cancer hospitals remained substantially lower than the national hospital average, which ranged from 66 to 79% during the study period.

**TABLE 8:** Resistance proportions (%) of *Acinetobacter baumannii* to antimicrobial agents.

Antimicrobial agent	2016	2017	2018	2019	2020	2021	2022	2023
AMK	4.3	6.1	6.5	10.4	12.3	11.8	9.7	15.3
GEN	11.7	13.4	12.9	18.9	16.9	17.4	17.7	23.2
TOB	9.2	11.7	11.3	9.5	10.7	12.4	9.9	14.1
SAM	13.3	13.3	9.4	12.2	12.8	13.8	15.4	16.0
TZP	8.2	7.4	9.2	13.7	14.0	13.9	13.6	19.1
CSL	22.8	4.2	9.5	13.5	10.3	12.8	12.9	20.4
CRO	14.9	14.7	11.0	16.8	19.0	20.9	17.3	18.7
CTX	7.0	9.2	9.6	13.7	17.5	10.3	10.0	10.1
CAZ	9.3	12.4	11.8	15.5	13.9	13.5	14.0	19.3
FEP	9.3	12.3	12.9	13.6	14.2	14.0	14.4	17.5
IPM	9.6	10.9	11.6	14.4	14.3	13.1	14.3	18.4
MEM	4.7	7.8	8.2	13.7	14.7	13.8	14.9	18.8
CIP	11.2	14.4	13.3	14.7	16.2	14.9	15.4	19.2
LVX	8.5	10.1	10.1	13.1	13.7	12.1	13.3	17.2
POL	7.5	1.3	0.0	3.3	0.0	2.0	1.9	1.3
MNO	27.4	21.4	8.4	8.7	8.1	7.1	9.3	8.4
TGC	1.8	0.7	0.6	0.8	0.5	0.8	0.6	0.6

AMK, amikacin; GEN, gentamicin; TOB, tobramycin; SAM, ampicillin-sulbactam; TZP, piperacillin-tazobactam; CSL, cefoperazone-sulbactam; CRO, ceftriaxone; CTX, cefotaxime; CAZ, ceftazidime; FEP, cefepime; IPM, imipenem; MEM, meropenem; CIP, ciprofloxacin; LVX, levofloxacin; POL, polymyxin B; MNO, minocycline; TGC, tigecycline.

### 3.9 Antimicrobial resistance patterns in key Gram-positive pathogens

#### 3.9.1 Staphylococcus aureus

The overall resistance proportions of Staphylococcus aureus to various antimicrobial agents are summarized in Table 9.

**TABLE 9:** Resistance proportions (%) of *Staphylococcus aureus* to antimicrobial agents.

Antimicrobial agent	2016	2017	2018	2019	2020	2021	2022	2023
PEN	93.0	93.1	93.5	92.0	92.1	93.3	92.0	91.8
GEN	17.5	14.5	13.1	12.0	9.1	7.5	8.6	8.1
RIF	3.0	2.3	2.0	1.9	1.5	1.2	1.5	1.4
LVX	16.9	17.1	15.3	13.8	14.6	12.2	11.4	13.4
SXT	18.1	15.1	14.8	11.6	12.9	12.6	13.0	12.7
CLI	41.7	35.2	35.7	31.3	28.2	34.3	30.2	30.9
ERY	62.1	57.6	57.9	56.1	51.4	54.0	49.7	50.4
LNZ	0.0	0.0	0.0	0.0	0.0	0.0	0.0	0.0
VAN	0.0	0.0	0.0	0.0	0.0	0.0	0.0	0.0
TEC	0.0	0.0	0.0	0.0	0.0	0.0	0.0	0.0

PEN, penicillin; GEN, gentamicin; RIF, rifampin; LVX, levofloxacin; SXT, trimethoprim-sulfamethoxazole; CLI, clindamycin; ERY, erythromycin; LNZ, linezolid; VAN, vancomycin; TEC, teicoplanin.

##### 3.9.1.1 MRSA

As shown in Table 10, MRSA was nearly 100% resistant to penicillin throughout the study. Based on the Joinpoint regression analysis (Table 5), this remained statistically stable (APC = 0.14%, 95% CI: −0.03 to 0.31, *P* = 0.102). The resistance proportions to levofloxacin showed a continuous downward trend (APC = −7.40%, 95% CI: −10.48 to −4.15, *P* < 0.001), whereas clindamycin remained statistically stable (2016–2019: APC = −7.25%, 95% CI: −17.04 to 3.29, *P* = 0.099; 2019–2023: APC = 0.77%, 95% CI: −9.01 to 12.71, *P* = 0.556). Moreover, MRSA resistance to erythromycin and trimethoprim-sulfamethoxazole decreased significantly from 2016 to 2019 (erythromycin: APC = −5.41%, 95% CI: −9.71 to −2.14, *P* = 0.003; trimethoprim-sulfamethoxazole: APC = −20.27%, 95% CI: −35.90 to −8.50, *P* = 0.004) before stabilizing (erythromycin 2019–2023: APC = −1.11%, 95% CI: −3.71 to 3.67, *P* = 0.653; trimethoprim-sulfamethoxazole 2019–2023: APC = 5.11%, 95% CI: −4.42 to 29.61, *P* = 0.290), with trimethoprim-sulfamethoxazole dropping overall from 16.6% in 2016 to 9.5% in 2023. Importantly, however, MRSA was not resistant to linezolid, vancomycin, or teicoplanin.

**TABLE 10:** Resistance proportion (%) of MRSA to antimicrobial agents.

Antimicrobial agent	2016	2017	2018	2019	2020	2021	2022	2023
PEN	99.5	98.3	99.7	99.6	99.7	99.8	99.8	100.0
GEN	18.9	16.6	15.4	11.9	10.5	7.0	11.6	9.4
RIF	8.3	5.2	4.8	4.5	3.6	3.4	3.5	3.0
LVX	30.2	31.8	26.7	20.1	22.7	20.0	19.0	19.3
SXT	16.6	13.0	14.3	7.7	8.6	9.4	12.6	9.5
CLI	65.2	55.4	57.6	49.7	48.9	56.4	51.1	51.2
ERY	83.4	80.0	76.2	70.8	67.8	72.1	70.2	66.5
LNZ	0.0	0.0	0.0	0.0	0.0	0.0	0.0	0.0
VAN	0.0	0.0	0.0	0.0	0.0	0.0	0.0	0.0
TEC	0.0	0.0	0.0	0.0	0.0	0.0	0.0	0.0

PEN, penicillin; GEN, gentamicin; RIF, rifampin; LVX, levofloxacin; SXT, trimethoprim-sulfamethoxazole; CLI, clindamycin; ERY, erythromycin; LNZ, linezolid; VAN, vancomycin; TEC, teicoplanin.

#### 3.9.2 Enterococcus faecium

As shown in Table 11, the resistance proportions of *E. faecium* to various antibiotics fluctuated over the study period. Based on the Joinpoint regression analysis (Table 5), between 2016 and 2019, resistance of *E. faecium* to quinolone antibiotics significantly declined (ciprofloxacin: APC = −2.57%, 95% CI: −5.93 to −0.08, *P* = 0.040; levofloxacin: APC = −2.07%, 95% CI: −3.90 to −0.91, *P* < 0.001); however, starting in 2019, levofloxacin resistance significantly rebounded (APC = 2.04%, 95% CI: 1.29–3.39, *P* < 0.001), while ciprofloxacin remained stable (APC = 1.55%, 95% CI: −0.15 to 5.21, *P* = 0.076), reaching 82.1% and 81.3%, respectively. Resistance to ampicillin remained stable over the study period (2016–2020: APC = −0.91%, 95% CI: −3.54 to 0.46, *P* = 0.139; 2020–2023: APC = 1.83%, 95% CI: −0.05 to 4.64, *P* = 0.057), fluctuating within a narrow range of 77.2–82.4%. Resistance to high-level gentamicin remained statistically stable despite numerically fluctuating from 21.2% in 2016 to 37% in 2019, then declined, reaching 33.7% in 2023 (APC = 7.08%, 95% CI: −2.19 to 17.37, *P* = 0.146). Regarding streptomycin, resistance significantly rose from 13.2% in 2016 to a peak in 2018 (2016–2018: APC = 41.45%, 95% CI: 12.84–74.02, *P* < 0.001), then stabilized (2018–2023: APC = −2.80%, 95% CI: −16.42 to 2.51, *P* = 0.236), oscillating between 22.6 and 26.3%. Of particular concern, low but statistically stable resistance to linezolid (2016–2018: APC = −69.78%, 95% CI: −89.14 to 25.60, *P* = 0.128; 2018–2023: APC = 51.57%, 95% CI: −15.19 to 416.19, *P* = 0.092), vancomycin (2016–2020: APC = −18.11%, 95% CI: −70.93 to 98.89, *P* = 0.278; 2020–2023: APC = 93.07%, 95% CI: −19.17 to 438.32, *P* = 0.084), and teicoplanin (APC = 29.67%, 95% CI: −18.37 to 108.64, *P* = 0.253) emerged during the surveillance period, with proportions in 2023 of 0.7, 2.2, and 1.4%, respectively, suggesting the potential emergence of resistance to last-resort agents.

**TABLE 11:** Resistance proportion (%) of *Enterococcus faecium* to antimicrobial agents.

Antimicrobial agent	2016	2017	2018	2019	2020	2021	2022	2023
CIP	82.4	82.2	81.2	76.2	77.6	80.4	80.9	82.1
AMP	81.8	80.6	80.0	79.2	77.2	82.1	82.4	81.7
LVX	79.5	79.6	76.9	74.7	77.0	78.8	80.2	81.3
RIF	55.6	59.5	68.6	64.9	67.1	79.6	81.3	59.7
GEH	21.2	25.8	28.3	37.0	25.2	38.8	38.0	33.7
STH	13.2	18.0	28.2	27.8	19.5	22.6	26.3	22.9
LNZ	1.9	0.5	0.1	0.4	0.3	1.1	1.2	0.7
VAN	0.5	0.8	0.8	0.4	0.2	1.0	1.2	2.2
TEC	0.0	2.9	0.7	1.2	3.6	3.0	1.9	1.4

CIP, ciprofloxacin; AMP, ampicillin; LVX, levofloxacin; RIF, rifampin; GEH, gentamicin high level; STH, streptomycin high level; LNZ, linezolid; VAN, vancomycin; TEC, teicoplanin.

#### 3.9.3 Enterococcus faecalis

Over the 8-year study period, as detailed in Table 12, *E. faecalis* exhibited divergent resistance trends. According to the Joinpoint regression analysis (Table 5), fluoroquinolone resistance remained statistically stable (ciprofloxacin 2016–2021: APC = 8.23%, 95% CI: −0.43 to 26.30, *P* = 0.060; 2021–2023: APC = −3.41%, 95% CI: −16.96 to 10.11, *P* = 0.755; levofloxacin: APC = 2.46%, 95% CI: −1.41 to 6.50, *P* = 0.191), despite numerical fluctuations. High-level gentamicin resistance significantly increased from 16.2 to 28.7% (APC = 8.78%, 95% CI: 6.23–11.43, *P* < 0.001), indicating growing challenges for combination therapy. In contrast, ampicillin resistance remained consistently low (APC = 0.16%, 95% CI: −10.60 to 12.57, *P* = 0.969), and linezolid resistance stayed minimal and stable (APC = 1.67%, 95% CI: −9.23 to 14.14, *P* = 0.721). Vancomycin resistance was rare and statistically stable (2016–2018: APC = −21.96%, 95% CI: −45.08 to 20.29, *P* = 0.348; 2018–2023: APC = 21.87%, 95% CI: −8.50 to 77.90, *P* = 0.081), while teicoplanin resistance remained statistically stable (APC = −17.30%, 95% CI: −44.31 to 24.28, *P* = 0.370) despite numerically dropping from 2.9% in 2016 to 0.4% in 2023, potentially reflecting effective antibiotic stewardship or reduced selective pressure.

**TABLE 12:** Resistance proportions (%) of *Enterococcus faecalis* to antimicrobial agents.

Antimicrobial agent	2016	2017	2018	2019	2020	2021	2022	2023
RIF	38.8	48.9	49.0	56.3	56.3	65.8	72.0	60.6
CIP	27.4	26.8	29.1	31.0	37.9	39.4	34.8	36.8
LVX	24.1	22.9	22.2	22.7	28.0	24.7	24.6	28.4
GEH	16.2	16.5	18.3	19.2	23.1	24.1	24.9	28.7
STH	12.4	11.9	22.9	21.7	24.6	22.1	23.7	25.8
AMP	3.8	3.1	3.4	2.4	2.1	3.2	5.2	2.8
LNZ	3.8	2.3	2.1	2.6	3.6	2.8	2.8	3.4
TEC	2.9	0.8	0.0	1.0	1.2	1.5	0.1	0.4
VAN	0.2	0.2	0.1	0.2	0.2	0.3	0.2	0.4

RIF, rifampin; CIP, ciprofloxacin; LVX, levofloxacin; GEH, gentamicin high level; STH, streptomycin high level; AMP, ampicillin; LNZ, linezolid; TEC, teicoplanin; VAN, vancomycin.

## 4 Discussion

This multicenter surveillance study spanning 23 cancer centers in China (2016–2023) provides a comprehensive overview of AMR in cancer patients. Contrary to the prevailing assumption that immunocompromised patients have higher AMR proportions, we observed a paradoxical yet reassuring pattern: resistance prevalence in this population was markedly lower than national proportions reported in general hospitals. This gap highlights the need for population-specific surveillance to guide empirical therapy and stewardship.

Our data confirm a distinct bacterial epidemiology in oncology. The Gram-negative-to-Gram-positive ratio (G^–^:G^+^) was 7.5:2.5, which is higher than the general-hospital average. However, this ratio declined gradually over time as Gram-positive isolates increased. This trend diverges from CHINET data, which show a slight rise in Gram-negative proportions (Hu et al., 2022). The divergence is likely multifactorial. Cancer-related immunosuppression, particularly T-cell dysfunction, has been associated with an increased susceptibility to Gram-positive pathogens such as coagulase-negative staphylococci and enterococci (Perfect et al., 2014). The frequent use of indwelling catheters and other invasive devices in oncology care may further elevate the risk of such infections (van den Bosch et al., 2021). By contrast, it has been proposed that more intensive broad-spectrum antibiotic use in general hospitals may exert different selective pressure on the microbial flora, potentially favoring Gram-negative bacilli (Ueda et al., 2023). The observed rise in Gram-positive isolates in our cohort could also be related to increased prophylactic anti-Gram-positive antibiotic use and strengthened infection-control measures targeting Gram-negative organisms in cancer centers (van de Wetering et al., 2013).

A particularly striking finding is the much lower carbapenem resistance in cancer hospitals compared with national averages. While carbapenem resistance among *K. pneumoniae* in general hospitals ranged from 22.6 to 23.4% during the study period (Guo et al., 2025), proportions in our cancer cohort remained below 2%. Resistance in *P. aeruginosa* and *A. baumannii*, though increasing, also remained substantially below national levels. Notably, *A. baumannii* exhibited sustained high susceptibility to polymyxin B throughout the study period, with resistance proportions remaining low (0.0–7.5%), reinforcing its critical role as a last-resort therapeutic option for managing multidrug-resistant *Acinetobacter* infections in oncology settings. Several oncology-specific factors may explain this: strict stewardship programs limiting carbapenem use as last-line agents reduce selective pressure (Itoh et al., 2024); the predominant infection syndromes differ, with more bloodstream infections (often Enterobacteriaceae) in cancer patients versus more respiratory infections due to non-fermenters (e.g., *A. baumannii*) in general hospitals (Pathoor et al., 2025; Xu et al., 2025); early active screening and contact precautions in cancer centers likely curb horizontal transmission of carbapenem-resistant organisms (Timsit et al., 2019); and general-hospital intensive care units, with higher acuity and longer stays, create conditions conducive to amplification and spread of multidrug-resistant organisms (Laxminarayan et al., 2020).

Despite lower resistance to key drugs such as carbapenems, the emergence of resistance to last-resort agents (e.g., linezolid and vancomycin) in *E. faecium* is a sentinel warning. This may reflect unique selective pressures within the oncologic microenvironment. Evidence suggests chemotherapy can disrupt gut commensals, reduce colonization resistance, and damage mucosal barriers, facilitating bacterial translocation (Ubeda et al., 2010; Taur et al., 2012). It has also been reported in previous studies that certain chemotherapies might activate bacterial SOS responses, which are theoretically thought to contribute to mutation and horizontal gene transfer, potentially fostering *de novo* resistance (Taur et al., 2012). This creates a vicious cycle: neutropenic fever requires immediate broad-spectrum antibiotics (Carratala et al., 1996; Perez et al., 2014), which further erode the microbiome, select resistant mutants, and may necessitate even broader coverage for subsequent infections (Rashidi et al., 2020). The low but detectable glycopeptide resistance observed in our data may represent an early signal of combined selective pressures from chemotherapy and antibiotic exposure.

Overall, the relatively low resistance proportions—especially to carbapenems—are encouraging and potentially reflecting effective infection control and stewardship, although causal inference is constrained by the absence of antibiotic consumption data. However, pathogen-specific challenges persist, including rising fluoroquinolone resistance in *E. faecalis* and the concerning (albeit low) emergence of last-resort resistance in *E. faecium*. These trends reinforce the notion that general hospital surveillance alone is insufficient to guide empirical therapy in oncology. The unique bacterial ecology and resistance dynamics in cancer patients, therefore, mandate dedicated monitoring systems. This study provides a foundational framework for such a network in China, filling a critical data gap and supporting tailored anti-infective guidelines for this vulnerable population.

A major strength of this study is its rigorous, homogeneous data-collection methodology. All participating centers followed standardized protocols, and data were validated using the CARSS web tool and independent third-party verification, ensuring objectivity and reliability.

## 5 Limitations

Several limitations must be acknowledged. First, and as a major limitation of this study, the retrospective design precluded establishing causal links between prescribing practices and observed resistance trends. While lower resistance proportions may be associated with enhanced infection control and antimicrobial stewardship, causal inference is limited by the lack of specific antibiotic consumption data. Although this issue was anticipated during the study design phase, systematically integrating such data proved unfeasible. Beyond the confidentiality of patient-level antibiotic and chemotherapy utilization, accurately evaluating these links across 23 provinces would require adjusting for complex macroscopic confounders, including distinct hospital operational models, diverse regional health insurance policies, and significant variations in local antimicrobial stewardship practices. Without these multidimensional data, it is impossible to directly link specific regimens to resistance patterns. Second, the essential difference in AMR profiles between patients with hematological malignancies and those with solid tumors is a major confounder in interpreting our overall data. Initially, we aimed to perform a subgroup analysis by tumor type; however, the lack of granular patient-level data precluded this. The CARSS database does not mandate the upload of specific clinical diagnoses. Furthermore, attempting to deduce tumor types based on admitting wards or departments proved completely unfeasible: due to specific hospital operational conditions and bed management, patients are frequently co-managed across discrete services, with cross-admission proportions (e.g., solid tumor patients in hematology wards and vice versa) exceeding 50% in some participating centers. Consequently, any ward-based classification would introduce severe misclassification and bias.

Third, from a microbiological perspective, the sputum predominance (44–54% of isolates) in our dataset may reflect bacterial colonization rather than true infection. Due to the absence of specific clinical diagnostic criteria to distinguish infection from colonization, the AMR proportions reported in this study may be underestimated. Thus, resistance proportions reported here may underestimate true infection-related resistance, particularly for pathogens with high colonizing potential (e.g., *A. baumannii, P. aeruginosa*). This is a common limitation inherent in large-scale national surveillance data in China, such as those from CHINET and CARSS. Fourth, our surveillance dataset relies exclusively on phenotypic antimicrobial susceptibility testing. Because molecular characterization was not performed, we cannot identify the specific underlying mechanisms for the reported carbapenem resistance profiles, such as differentiating between carbapenemase production, porin loss, or efflux pump overexpression. Fifth, regarding geographic representativeness, although our network covers 20 provincial-level regions representing approximately 1.1 billion people, it does not encompass all 34 provinces, potentially limiting generalizability. Finally, some cancer patients receive care in non-specialist hospitals, and their data were not captured, introducing a potential selection bias. Future prospective studies should integrate clinical, microbiological, pharmacological, and administrative data to elucidate these complex interactions and further refine treatment strategies.

## Data Availability

The original contributions presented in this study are included in this article/supplementary material, further inquiries can be directed to the corresponding authors.
